# Alternative Strategies for Managing Dental Black Stains: A Systematic Review

**DOI:** 10.3390/dj14100638

**Published:** 2026-10-04

**Authors:** Omaima Boukhancha, Manal Amimer, Sanaa Chala

**Affiliations:** 1 Department of Conservative Dentistry and Endodontics, Faculty of Dental Medicine, Mohammed V University, Rabat 10112, Morocco; s.chala@um5r.ac.ma; 2 Department of Periodontology, Faculty of Dental Medicine, Mohammed V University, Rabat 10112, Morocco; manal_amimer@um5.ac.ma; 3 Mohammed V Military Teaching Hospital, Rabat 10100, Morocco; 4 Laboratory of Biostatistics, Clinical and Epidemiological Research, Faculty of Medicine and Pharmacy, Mohammed V University, Rabat 10100, Morocco

**Keywords:** systematic review, black stain, chromogenic bacteria, extrinsic tooth discoloration, chromogenic biofilm, alternative treatment, adjunctive treatment, probiotics, photodynamic therapy, recurrence

## Abstract

**Background/Objectives:** Dental black stains (BSs) are a form of extrinsic tooth discoloration of bacterial origin, with a prevalence ranging from 2.4% to 20%. As scaling and polishing remain the standard of care, recurrence is frequent. Therefore, this systematic review assessed alternative or adjunctive treatments, such as probiotics, chemical agents, and photodynamic therapy, for managing BSs. The primary objective was to evaluate clinical changes in BS recurrence and severity, while the secondary objective was to analyze microbiological changes. **Methods:** Conducted following PRISMA 2020 guidelines and registered on PROSPERO (CRD420261357137), this review searched PubMed, Cochrane Library, Scopus, Web of Science, and Dentistry & Oral Sciences Source through EBSCOhost as well as clinical trial registers (ClinicalTrials.gov and ISRCTN) and grey literature, up to 30 April 2026, with no language or date restrictions. We included randomized and non-randomized clinical trials. Two independent reviewers performed study selection and data extraction; a third resolved disagreements. We assessed risk of bias using RoB 2 and ROBINS-I V2, and quality of evidence using GRADE. **Results:** Of 716 records identified, eight clinical trials (five randomized clinical trials and three non-randomized clinical trials were included), with 250 patients enrolled and 227 assessed at follow-up. Four intervention categories were investigated: probiotics, antimicrobial mouthwashes (0.1%, 0.12% chlorhexidine and 0.1% chlorine dioxide), enzyme-containing toothpaste, and antimicrobial photodynamic therapy (aPDT). Probiotics may reduce BS indices, though the effect was inconsistent over time. In a single small, randomized study, chlorine dioxide showed a short-term reduction in bacterial viability greater than chlorhexidine. aPDT and enzyme-containing toothpaste did not show a significant benefit in the included studies. High risk of bias, heterogeneity, and the small sample prevented quantitative synthesis. **Conclusions:** Evidence supporting alternative strategies for managing recurrent dental BSs or reducing chromogenic bacterial load remains insufficient. Larger RCTs with standardized outcomes and longer follow-up are needed.

## 1. Introduction

Black stains (BSs) are a form of extrinsic dental discoloration characterized by darkly pigmented deposits, with high adherence to the tooth surface. This condition may occur at any age, but it is more common in children and affects both sexes, with a reported prevalence ranging from 2.4% to 20% [1]. Clinically, BS appears as discontinuous light brown to black dots or dark lines located along the cervical third of buccal and/or lingual surfaces. In more severe cases, they may extend to cover large areas of the crown and even involve pits and fissures. When newly formed, the deposits cannot be detected by probing; however, as they mature, they develop a granular consistency that can be detected with a probe. These discolorations negatively affect dental aesthetics, which may in turn impact patients’ psychological well-being and self-esteem, particularly children, who may experience bullying, potentially leading to increased psychosocial distress. Other discolorations should be differentiated from BSs: On one hand, intrinsic discolorations result from the incorporation of chromogenic substances into enamel and dentin during tooth formation or after eruption (fluorosis, chemical changes due to tetracycline…) [2]. On the other hand, extrinsic discoloration includes tobacco staining, which may have a similar color but differs in appearance and distribution, and food staining, which typically presents as less dense deposits on the surface of the tooth [3].

The pigment in BSs is thought to consist of an insoluble ferric compound, most likely ferric sulfide, resulting from the interaction between bacterial hydrogen sulfide and iron present in saliva or gingival fluid [4,5]. Microbiological investigations have shown that BSs are associated with a specific oral biofilm composition, with *Actinomyces* spp. frequently detected, along with other species such as *Aggregatibacter actinomycetemcomitans*. Several other factors are suspected in the literature to influence the development and severity of BSs, including age, dietary habits, oral hygiene practices, socioeconomic status, and iron supplementation [3].

Currently, professional prophylaxis (scaling and polishing) remains the standard approach for managing BSs. However, recurrence may occur even with good oral hygiene [6]. Moreover, this intervention does not address the underlying microbial etiology of the condition, underscoring the lack of a definitive and sustainable therapeutic strategy [1,7]. In response to these limitations, treatments aim to reduce recurrence beyond mechanical removal or to reduce the bacterial load of chromogenic bacteria, including chemical agents, probiotics, and photodynamic therapy. Regarding chemical agents, toothpastes and mouthwashes are the most commonly investigated formulations in the literature. For probiotics, defined by the World Health Organization and the Food and Agriculture Organization as “live microorganisms which, when administered in adequate amounts, confer a health benefit on the host”, have also attracted growing interest [3]. In addition, photodynamic therapy has emerged as another non-invasive antimicrobial approach. This technique is based on the principle that non-toxic photosensitizers can be preferentially localized in specific tissues and subsequently activated by light at an appropriate wavelength to generate singlet oxygen and free radicals that induce bacterial cell death [8].

Hence, this systematic review aims to assess the effectiveness of alternative or adjunctive modalities, including chemical agents, probiotics, and photodynamic therapy, in reducing the recurrence or the severity of extrinsic dental black stains of bacterial origin.

## 2. Methods

### 2.1. Protocol and Registration

This systematic review followed the Preferred Reporting Items for Systematic Reviews and Meta-Analyses (PRISMA) recommendations [9] (File S1). The research was registered with PROSPERO under the registration number CRD420261357137.

### 2.2. Eligibility Criteria

#### 2.2.1. Inclusion Criteria

Studies were selected based on the following PICOS (Population, Intervention, Comparator, Outcome, Study design) framework:

**Population**: Trials involving children and adults (both genders) were included if they explicitly had black stains (BSs) as extrinsic dental discolorations of bacterial etiology, based on clinical appearance and localization. Studies utilizing subjects without black stains as a negative control group were also eligible.

**Intervention**: Alternative or adjunctive intervention other than mechanical scaling and/or polishing (a baseline professional prophylaxis can be a precursor, especially for clinical outcomes).

**Comparators**: Valid reference controls consisted of standard professional hygiene procedures, other alternative treatment, placebo, or no treatment.

**Outcomes**:

- Primary Outcomes: Stain recurrence and clinical severity. This domain focuses on the intervention’s efficacy in reducing black stain recurrence. It includes measures of stain presence/absence (binary), standardized clinical scoring indices (Pigmented Plaque Index [Silness and Löe], Lobene Stain Index [modified by Gründermann], and Shourie Index), time to BS reappearance, and changes in stain area.
- Secondary Outcomes: Microbiological changes, including bacterial counts, viability, colonization levels, and salivary bacterial load. These outcomes were evaluated to explore underlying biological mechanisms, distinct from clinical efficacy.

**Study Design**: Clinical trials (randomized and non-randomized) were included. For studies combining in vivo and in vitro components, only data derived from the in vivo clinical trial segment were eligible for extraction.

Language and Follow-up: No categorical minimum follow-up period was established. No language restrictions were applied; articles not published in English were translated using DeepL to facilitate screening and data extraction.

#### 2.2.2. Exclusion Criteria

Studies were excluded if they met any of the following criteria: (1) laboratory, animal, or in vitro-only studies; (2) descriptive reports including case reports, case series, narrative reviews, editorials, and letters; (3) studies focusing on dental discolorations caused by etiologies other than chromogenic bacteria (e.g., tobacco-induced, metal-induced, or drug-related staining); (4) conference abstracts or unpublished manuscripts where insufficient data were available for methodological assessment; and (5) studies for which the full text could not be retrieved after a contact attempt to the corresponding author.

### 2.3. Information Sources

The literature search was conducted from January to April 2026 across different electronic databases, including PubMed (MEDLINE), Scopus, Clarivate Analytics Web of Science, Dentistry & Oral Sciences Source (EBSCOhost), and the Cochrane Central Register of Controlled Trials.

We conducted a simple initial search to identify keywords and equivalent controlled vocabulary, such as Medical Subject Headings (MeSH). Moreover, a search was conducted on OpenGrey and Google Scholar.

In addition, clinical trial registries, such as the ISRCTN registry and ClinicalTrials.gov, were screened for ongoing studies.

To ensure comprehensive coverage, backward and forward snowballing techniques were used and authors were contacted when necessary. The last database update was on 30 April 2026.

### 2.4. Search Strategy

The search strategy/equation used in the databases was as follows:

((“black stain*” OR “dental black stain*” OR “extrinsic black stain*” OR “chromogenic stain” OR “chromogenic bacteria”) AND (treatment OR therapy OR removal OR management OR scaling OR polishing OR prophylaxis OR probiotic* OR toothpaste* OR mouthwash* OR “chemical agent*” OR photodynamic OR “photodynamic therapy” OR “PDT” OR “photoactivated disinfection” OR “photochemotherapy”)).

No restrictions on language or year of publication were made. The search strategy is provided in Appendix A Table A1.

### 2.5. Selection Process

Study selection was performed using the Rayyan web-tool (Rayyan Systems Inc., Cambridge, MA, USA) [10], by two independent reviewers (O.B and M.A), blinded, to eliminate duplicates and screen titles and abstracts, followed by full-text assessment of potentially relevant studies. These reports were classified as excluded, included, or maybe.

Comparative analyses were conducted between lists of pertinent studies, and in case of disagreement, a third investigator (S.C.) made the final decision.

### 2.6. Data Collection Process

The selected studies underwent independent data extraction by two review authors (O.B. and M.A.). Both reviewers used a standardized sheet that included the first author’s name, year of publication, country, study design type, time points, study groups, population characteristics (gender and age range), measured outcomes, and results. Disagreements were resolved through discussion with a third reviewer (S.C.), and references were managed in Zotero.

All compatible time points reported in each study (e.g., baseline, 1 month, 3 months, 6 months, and 12 months) were extracted to capture both short and long-term efficacy.

### 2.7. Risk of Bias Assessment

The quality of randomized controlled studies included in the systematic review was assessed using the revised Cochrane Risk-of-Bias tool for randomized trials (RoB 2) (version of 22 August 2019) [11]. This tool assesses five domains: bias arising from the randomization process; bias due to deviations from intended interventions; bias due to missing outcome data; bias in the measurement of the outcome; and bias in the selection of the reported result. Each domain includes a series of signaling questions that guide the judgment of risk of bias as low risk, some concerns, or high risk.

For non-randomized studies, the risk of bias was assessed using the ROBINS-I V2 (Risk Of Bias In Non-randomized Studies of Interventions, Version 2) tool (version 20 November 2025) [12]. This tool includes an initial preliminary assessment to determine whether the study is suitable for evaluation. If appropriate, six domains are assessed: bias due to confounding, bias in the classification of interventions, bias in the selection of participants into the study, bias due to missing data, bias in the measurement of outcomes, and bias in the selection of the reported result. Each domain is judged to be at low, moderate, serious, or critical risk of bias.

Risk of bias assessments were performed for the primary and secondary outcomes of interest; an overall risk of bias judgment was then assigned to each study based on the algorithm across domains, which was performed by the two reviewers (O.B and M.A). Each disagreement was resolved through discussion and consensus, and the judgment of a third reviewer (S.C.) was sought to ensure the results were valid.

### 2.8. Effect Measures

To evaluate comparative treatment effects, outcomes were categorized as binary or continuous. For binary outcomes (presence or absence of dental black stains), the primary effect measure of interest was the risk ratio (RR) with 95% confidence intervals (CIs). For continuous outcomes evaluated via black stain scores such as the Lobene Stain Index, Shourie Index, or Pigmented Plaque Index, the primary effect measure was the mean difference (MD) with 95% CIs.

### 2.9. Synthesis Methods

Due to substantial clinical and methodological heterogeneity among the included studies, a quantitative meta-analysis was deemed inappropriate. Consequently, we performed a structured narrative synthesis in accordance with the Synthesis Without Meta-analysis (SWiM) reporting guidelines [13]. Studies were grouped a priori by intervention category to facilitate comparisons: (1) probiotic interventions, (2) antimicrobial mouthwashes and chemical agents, and (3) antimicrobial photodynamic therapy (aPDT). For each category, we synthesized both primary clinical outcomes (stain recurrence and severity) and secondary microbiological outcomes (including chromogenic bacterial load).

To standardize clinical evaluation, follow-up intervals were categorized into short-term (≤3 month) and long-term (>3 months). Attrition rates and missing outcome data were extracted for each trial; their potential impact was assessed through risk-of-bias evaluation (RoB 2 and ROBINS-I V2), specifically focusing on domains pertaining to missing data, while prioritizing available-case analyses and noting whether studies performed intention-to-treat (ITT) or per-protocol (PP) analyses. For studies providing sufficient data, we calculated standardized effect estimates to enhance comparability.

Because we did not perform a meta-analysis, we did not calculate statistical heterogeneity. Instead, we conducted a qualitative investigation of heterogeneity by examining variations in participant age, diagnostic criteria, and intervention protocols. Sensitivity analyses were performed by restricting the synthesis to studies with “low” or “some concerns” risk of bias to assess the robustness of our conclusions regarding clinical efficacy.

### 2.10. Certainty Assessment

The quality of evidence was evaluated using the Grading of Recommendations, Assessment, Development and Evaluation (GRADE) [14]. Randomized controlled trials (RCTs) start as high-quality evidence, whereas non-randomized controlled trials (NRCTs) start as low-quality evidence. Studies can be downgraded by one level if there are serious concerns or two levels if there are very serious concerns in any of five domains: risk of bias, inconsistency, indirectness, imprecision, or publication bias. Consequently, the quality of evidence for each outcome is classified into one of four categories: high (strong confidence that the true effect corresponds to the estimate), moderate (moderate confidence), low (limited confidence), and very low (very little confidence) [15].

## 3. Results

### 3.1. Study Selection Process

To select relevant studies for this systematic review, a structured literature search was conducted, as detailed in the flowchart (Figure 1). Initially, the search strategy identified 715 publications from databases and registers, of which 340 duplicates were removed. Following title and abstract screening, an additional 361 records were excluded, leaving 14 studies for full-text assessment. Of these, three were completed but unpublished, three others are ongoing trials, and one additional study was excluded as it evaluated a different intervention, specifically, scaling and polishing alone without any adjunctive intervention (Table S1). Thus, seven studies remained. One study was also identified through Google Scholar. Ultimately, eight studies met the inclusion criteria and were included in this systematic review. Of the included studies, six were published in English, and two in Italian.

Across the included studies, 250 participants were initially enrolled. However, attrition was reported in three studies, resulting in 227 participants available for analysis at subsequent follow-up time points. The reasons for attrition included loss to follow-up (*n* = 5), no longer meeting the study eligibility criteria (*n* = 1), non-compliance with the study protocol (*n* = 3), orthodontic treatment (*n* = 3), presence of caries (*n* = 7), and antibiotic therapy during the study period (*n* = 4), which led to exclusion from the analysis.

### 3.2. Characteristics of the Included Studies

Eight clinical trials included in this review investigated alternative or adjunctive treatments for dental black stains of bacterial origin. Of these, five were randomized, and three were non-randomized studies. Five studies were conducted in Italy [16,17,18,19,20], one in Indonesia [21], one in Spain [22], and one in Egypt [23]. The studies were published between 2010 and 2025 and involved a total of 250 patients enrolled and 227 analyzed. The detailed characteristics of the included studies are presented in (Table 1).

### 3.3. Assessment of Risk of Bias

Among the included randomized controlled trials, three outcomes were judged to be at high risk of bias (Figure 2): the presence or absence of black stains in the study by Bardellini et al. [18], the Shourie Index, and the prevalence of periodontal pathogens in the study by Carelli et al. [20]. This risk of bias is due to the deviation from the intended intervention and missing outcomes. In contrast, *Actinomyces* spp. bacterial viability in the study by Eunike et al. [21], the Lobene Stain Index in the study by Giannini et al. [17], and the modified Lobene index in the study by D’Errico et al. [19], were judged to have some concerns and this is due to the randomization process (D1) and selection of the reported result (D5).

For the three non-randomized studies (Bartolino et al. [16]; Albelda-Bernardo et al. [22]; and Wahba et al. [23]), the ROBINS-I V2 tool was utilized to evaluate bias across six domains (Figure 3). All three studies were categorized as having a serious overall risk of bias. Bias due to confounding (D1) was a predominant issue; this domain was rated as serious in all outcomes, reflecting the difficulty in controlling for baseline variations in oral hygiene and diet. Across the studies, the classification of interventions (D2) and the selection of participants (D3) ranged from low to moderate risk, suggesting that the study designs were clear in their intervention protocols. (D4) for bias due to missing data was judged to be low risk in Albelda-Bernado et al. [22]. and Wahba et al. [23] and moderate risk in Bartolino et al. [16]. Measurement of outcome (D5) was rated as moderate risk for four outcomes from two studies (Bartolino et al. [16] and Albelda-B et al. [22]) and low risk for two outcomes from the Bartolino et al. [16] and Wahba et al. [23] studies. For (D6) selection of the reported result, there was low risk in two studies and moderate risk in Bartolino et al. [16].

D1 represents bias due to randomization process, D2 represents bias due to deviations from intended interventions, D3 represents bias due to missing outcome, D4 represents bias in the measurement of outcomes, and D5 represents bias due to selection of the reported result.

D1 represents bias due to confounding, D2 represents bias in classification of interventions, D3 represents bias in selection of participants into the study, D4 represents bias due to missing data, D5 represents bias in measurement of outcomes, and D6 represents bias in selection of the reported result.

### 3.4. Level of Evidence

A narrative GRADE approach was used to assess the quality of evidence across the eight studies. The outcomes chosen were recurrence and bacterial load. The overall analysis ranged from low to very low (Table 2). The domains that downgraded the outcomes most were risk of bias, imprecision due to small samples, and indirectness for bacterial load.

## 4. Discussion

Some recent reviews on dental black stains have primarily mapped broad etiologic, microbiological, and management concepts. Specifically, in the scoping review by Kumar et al. [1] and the narrative review by Al-Shareef et al. [3], both authors mentioned management strategies mainly from case reports and in vitro studies. The present systematic review isolates clinical trials evaluating adjunctive interventions to professional scaling and polishing, focusing on three outcomes: clinical stain recurrence, severity, and chromogenic microbial shifts. Across the eight included studies, four distinct therapeutic categories were identified: probiotics, chlorhexidine or chlorine dioxide-based mouthwashes, dentifrices containing enzymes and salivary proteins, and antimicrobial photodynamic therapy.

Probiotics represented the most investigated approach. Two main bacteria were evaluated: *Lactobacillus reuteri* and *Streptococcus salivarius*.

For recurrence and severity of black stains, Bardellini et al. [18] and D’Errico et al. [19] found a reduction in the reappearance of black stains and the modified Lobene index, respectively, in the short term (2 to 3 months), for different molecules and durations. Giannini et al. [17] compared the same molecule for different durations of administration with an inter-group head-to-head comparison, which yielded a non-significant result. The three studies have a very low certainty of evidence due to their risk of bias and imprecision.

For bacterial load, Wahba N et al. [23] evaluated the effects of a combination of probiotics on the bacterial loads of *A. naeslundi* and *A. actinomycetemcomitans* after two weeks. Their results indicated a complete eradication of *A. actinomycetemcomitans* and a reduction in *A. naeslundii.* This study investigated outcomes over a short period and was classified as having a serious risk of bias due to confounding variables. In the literature, a case study by Yu JJ et al. [24] reported findings consistent with the local direct effect of probiotics, suggesting that, as an adjunct, applying probiotic powder to black-stained tooth surfaces facilitated the removal of stains, and observed no recurrence with brushing and flossing alone. The observed effect may be explained by mechanisms previously described in the literature; probiotics may exert antipathogenic action through direct chemical secretions, physical interactions, or immunomodulatory pathways; for example, *Lactobacillus reuteri* secretes specialized water-soluble antimicrobial molecules like reuterin and reutericyclin, which are highly stable because they resist lipolytic and proteolytic enzymes and remain active across a broad pH range. Other probiotics may physically co-aggregate with sulfur compound-producing oral pathogens, and lastly, they may stimulate the host’s immune response by enhancing barriers and modulating innate and adaptive immunities, such as through IgA and defensins [25,26,27].

For antimicrobial mouthwashes, Bartolino et al. [16] reported a reduction in the Pigmented Plaque Index up to 6 months while using 0.12% chlorhexidine, and shifts in microbiological profile while using chlorhexidine 0.1% and chlorine dioxide 0.1% were evaluated in this context. Eunike MC et al. [21] observed a reduction in the viability of *Actinomyces* sp. with both agents, with chlorine dioxide showing a greater reduction after 7 days of rinsing, and a mean difference of 0.20 (95% CI [0.05, 0.35]) with a *p* value of 0.012. However, the wide interval reflects low precision, a structural limitation attributable to the study’s small sample size. Another investigated approach involves enzyme-containing toothpastes enriched with salivary proteins. The rationale for these formulations lies in their potential to modulate the oral microbiota and enhance natural salivary defense mechanisms [28,29]. Carelli M et al. [20] evaluated this approach in a 14-week RCT and indicated that using an electric toothbrush may have played a greater role in reducing the Shourie Index than the toothpaste formulation, emphasizing the importance of mechanical plaque control. This observation contrasts with the findings of Prathap S. et al. [6], who reported no clear association between oral hygiene practices and the occurrence of black stains. These results suggest that the intervention’s role in preventing black stain recurrence remains uncertain and is likely secondary to mechanical plaque removal.

The final approach investigated was antimicrobial photodynamic therapy (aPDT), which was evaluated in a non-randomized clinical trial by Albelda-Bernardo et al. [22]. The addition of aPDT failed to delay recurrence timelines, resulting in an identical clinical comeback period. However, minor alterations in stain surface area and color were reported (observed in 38.7% of patients), and were highly variable, likely confounded by unmonitored dietary habits or routine oral hygiene practices [8]. Those studies showed low to very low certainty of evidence.

### 4.1. Clinical Relevance

From a clinical perspective, recurrent black stains should still be managed primarily through professional mechanical removal, with reinforcement of oral hygiene measures. Probiotics may be an adjunct, but their use cannot yet be recommended as standard protocol due to limited, heterogeneous evidence. Antimicrobial mouthwashes containing chlorine dioxide or chlorhexidine may help reduce bacterial load in the short term, and long-term use should be considered cautiously. Neither mouthwashes, photodynamic therapy, nor enzyme-based toothpaste currently has sufficient evidence to support their use against black stains.

### 4.2. Limitations

Several limitations must be acknowledged when interpreting the present findings. The overall quality of the available evidence remains limited. As presented in Figure 2 and Figure 3, the risk of bias across the included studies ranged from some concerns to high risk. In addition, the inclusion of a non-randomized clinical trial limits the strength of the available evidence. Small sample sizes, short follow-up periods, and substantial methodological heterogeneity in interventions, treatment duration, and outcome assessment methods also characterized most of the included studies. Consequently, the heterogeneity among the included studies and the limited number of high-quality randomized controlled trials did not permit a comprehensive meta-analysis of all investigated outcomes and the narrative quality of level of evidence of outcomes evaluated was low to very low using GRADE (Table 2). A marked geographical clustering was present, as five of the eight included clinical studies originated from a single country (Italy), which may limit the external validity and generalizability of the findings. Also, the inability to retrieve some reports may lead to the possible exclusion of unpublished evidence. In fact, direct communication with investigators of completed registered trials confirmed that at least one completed study remained unpublished due to statistically non-significant findings.

### 4.3. Research Priorities

Further studies are needed to investigate these approaches, especially probiotics with different strains, using well-designed randomized controlled trials with larger sample sizes, standardized diagnostic criteria for black stains, and longer follow-up periods to enable generalization of the results. There is also a need to harmonize outcome measures, since the included studies used different indices such as the Lobene index, Shourie Index, and Pigmented Plaque Index, and to use more objective outcomes to reduce bias.

## 5. Conclusions

In conclusion, the current evidence is insufficient to support the use of alternative or adjunctive interventions for managing black stains. Very low-certainty evidence suggests that some probiotic interventions may reduce black-stain recurrence or severity in the short term, but the effect is uncertain and has not been consistently demonstrated at longer follow up. Evidence for mouthwashes, enzyme-containing toothpaste, and photodynamic therapy is insufficient to establish a clinically meaningful effect on recurrence.

## Figures and Tables

**Figure 1:**
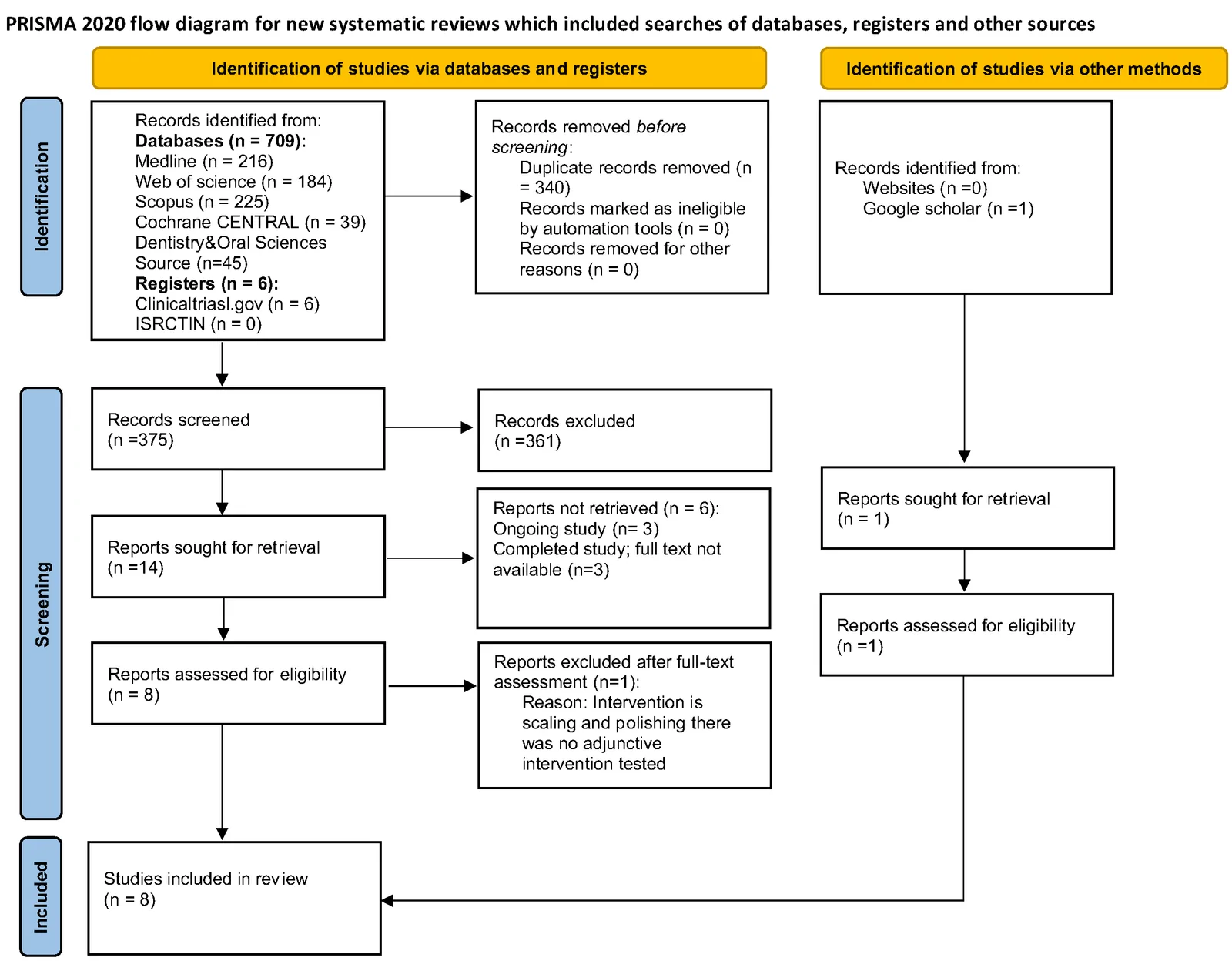
PRISMA flow diagram of the study search and identification.

**Figure 2:**
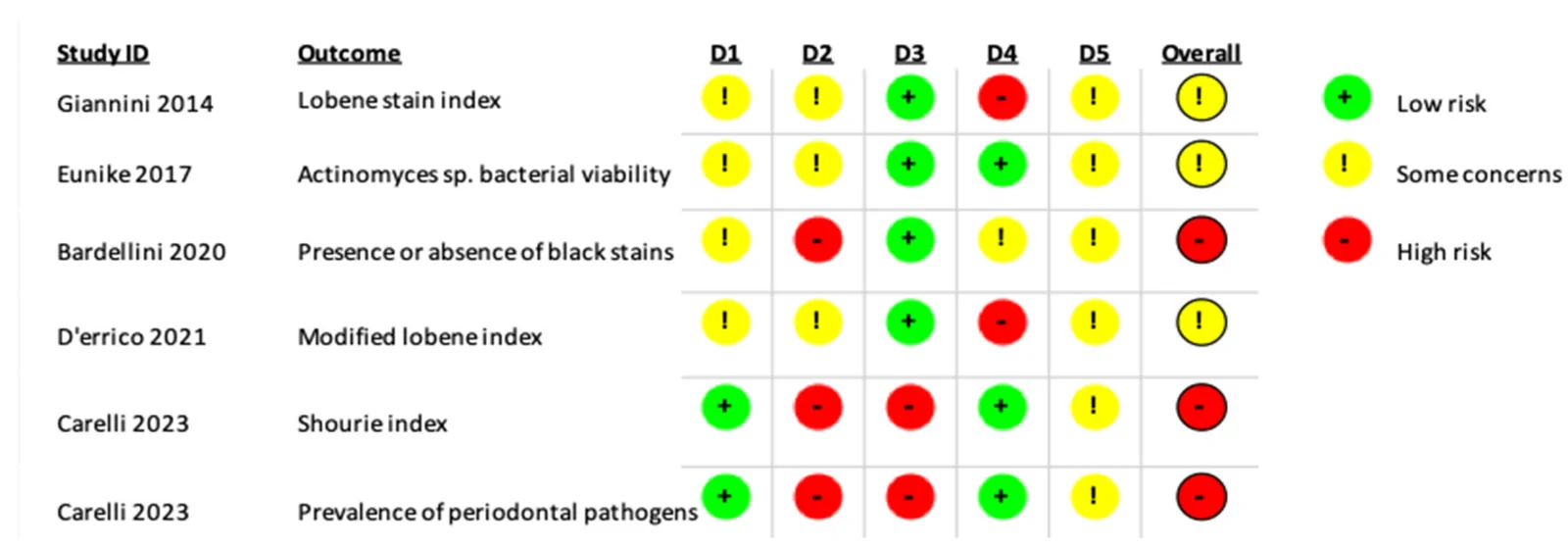
Assessment of risk of bias among randomized controlled trials for Giannini et al. [17], Eunike et al. [21], Bardellini et al. [18], D’Errico et al. [19] et Carelli et al. [20].

**Figure 3:**
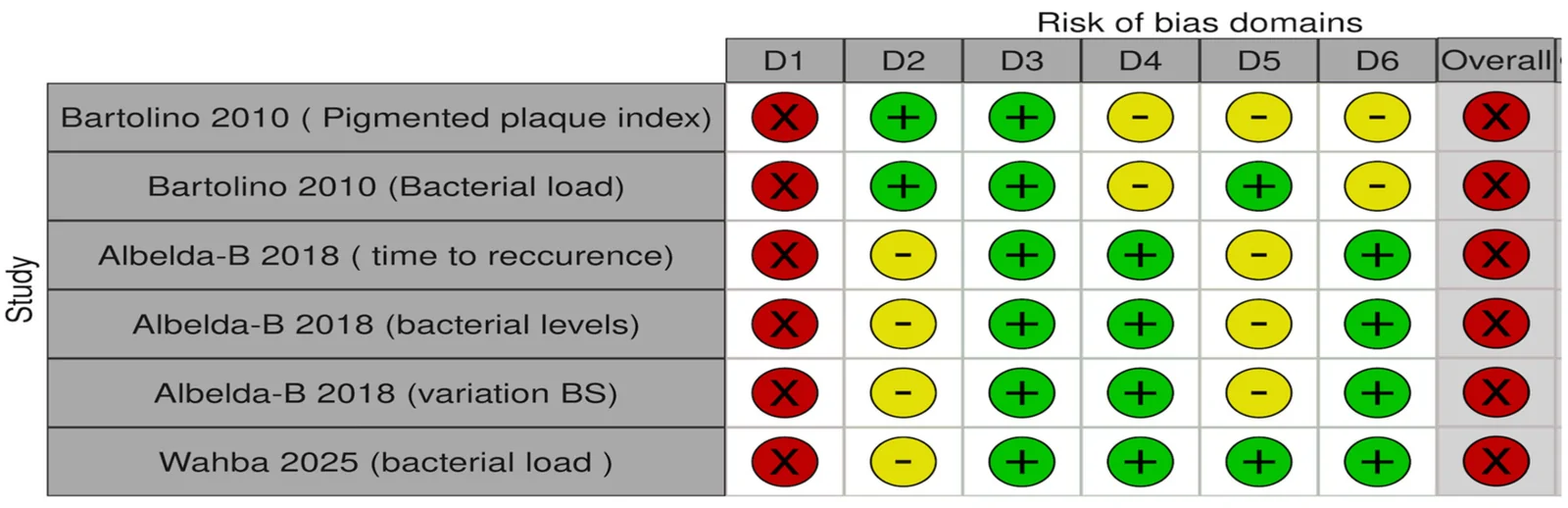
Risk-of-bias table for non-randomized studies for Bartolino et al. [16], Albelda-B et al. [22]. and Wahba et al. [23].

**Table 1:** Overview of the characteristics of the included studies.

First Author, Year of Publication, (Country)	Study Design	Participants	Study Groups	Follow-Up	Attrition	Measured Outcomes	Results
**Bartolino M** **2010 (Italy)** [16]	Non-randomized controlled clinical trial and microbiological analysis	-Enrolled: 63 patients 2–15 years F: 31 M: 32 -Analyzed: 63 patients	Professional cleaning and polishing, and fluoroprophylaxis for all groups. **Group 1:** No mouthwash was prescribed. **Group 2:** 0.05% sodium fluoride mouthwash used for 15 days every three months (for a total of two treatment cycles). **Group 3:** 0.12% chlorhexidine mouthwash for 15 days every three months (for a total of two treatment cycles).	-T_0_: 1st day -T_1_: 18th day -T_2_: 3 months -T_3_: 3 months and 18 days -T_4_: 6 months	*n* = 0	-Pigmented Plaque Index (Silness and Löe) -Bacterial count	**Pigmented Plaque Index (Silness and Löe, 1964) (percentage)** **Group 1:** T_0_: 18.45%; T_1_: 10.2%; T_2_: 12.58%; T_3_: 21.47%; T_4_: 11.18% **Group 2:** T_0_: 20.17%; T_1_: 7.77%; T_2_: 13.79%; T_3_: 7.55%; T_4_: 13.5% **Group 3:** T_0_: 20.86%; T_1_: 7.2%; T_2_: 7.06%; T_3_: 4.92%; T_4_: 6.02% **Reduction/increase in the number of colony-forming units (CFUs) on agar plates from patients’ plaque samples** Between T_0_ and T_1_: G1: +56%; G2: −52%; G3: −89% Between T_0_ and T_2_: G1: −36%; G2: −77%; G3: −74% Between T_1_ and T_2_: G1: −59%; G2: −52%; G3: +50%
**Giannini L** **2014 (Italy)** [17]	Randomized controlled clinical trial	-Enrolled: 10 patients 25–35 years F: 5 M: 5 -Analyzed: 10 patients	Both groups had scaling and polishing at T_0_. **Group A:** Treatment with a probiotic product containing *L. reuteri* for two months. **Group B:** The same product for one month only.	-T_0_: 1st day -T_1_: 1 month -T_2_: 4 months	*n* = 0	-Lobene Stain Index modified by Gründemann -Plaque Index (Sinless and Löe)	**Lobene Stain Index:** **Group A:** T_0_: 3–35%; T_1_: 0–6%; T_2_: 0–21% **Group B:** T_0_: 7–48%; T_1_:5%; T_2_: 0.9–42% **Plaque Index (Silness and Löe):** T_0_: GA: 5–42%; GB: 9–70% T_1_: GA: 2–35%; GB: 12–69% T_2_: GA: 5–63%; GB: 20–48% Limited improvement, with mostly non-significant changes in Group A and variable significance in Group B.
**Eunike MC** **2017 (Indonesia)** [21]	Randomized, single-masked clinical trial, and laboratory observation	-Enrolled: 16 patients 6–11 years F: 2 M: 14 -Analyzed: 16 patients	**Group 1:** 10 mL of experimental mouthwash containing 0.1% chlorine dioxide, used for 7 days, twice daily (after breakfast and before sleep), for 30 s each time. **Group 2:** Instructions to rinse in the same way with a mouthwash containing 0.1% chlorhexidine.	-T_0_: 1st day -T_1_: 7th day	*n* = 0	-*Actinomyces* sp. bacterial viability	**The mean values of *Actinomyces* sp. bacterial viability** **Group 1:** 1st day: 0.73 ± 0.11, 7th day: 0.40 ± 0.21 *p* value: 0.001 * Group 2: 1st day: 0.67 ± 0.18, 7th day: 0.54 ± 0.09 *p* value: 0.010 * The mean value differences in *Actinomyces* sp. bacterial viability between the two mouth rinses **Group 1:** 0.33 ± 0.17 **Group 2:** 0.13 ± 0.10 Mean difference 0.20 (95% CI [0.05, 0.35], *p* value: 0.012 *)
**Albelda-Bernardo M A** **2018 (Spain)** [22]	Non-randomized clinical trial and laboratory observation	-Enrolled: 38 patients F: 24 M 14 -Analyzed: 31 patients	**One study group:** Prophylaxis at T0, then follow-up until recurrence, then prophylaxis and antimicrobial phototherapy at 475 nm (aPT).	Monthly follow-up for an average period of 10.5 months	*n* = 7 -Reason for attrition: * lost to follow-up (*n* = 5) * no longer met the study inclusion requirements (*n* = 1) * failed to comply with the established protocol (*n* = 1)	-Time to reappearance of black stains -Variations in the area and color of black stains -Levels of colonization by *A. actinomycetemcomitans*, *P. gingivalis*, and *T. forsythia*	**Reappearance of BSs:** After dental prophylaxis only: 5.3 months After dental prophylaxis once again, followed by the application of aPT: 5.2 months *p* = 0.762 **Area of coverage of BSs pre- and post-aPT:** No significant change: 45.2% of patients Reduction: 38.7% of patients Increase: 16.1% of patients *p* = 0.008 * Modification of BS color due to aPT: Lighter than initial color: 26% of patients Returned to baseline color: 74% of patients *p* < 0.05 * Modification of bacterial levels in BSs due to aPT *T forsythia*: pre-aPT 83.9%; post-aPT 80.6% *P. gingivalis*: pre-aPT 29.03%; post-aPT 19.4% *A.actinomycetemcomitans*: Pre-aPT: 9.7%; post-aPT: 6.5% *p* > 0.05
**Bardellini E** **2020 (Italy)** [18]	Randomized controlled clinical trial	-Enrolled: 68 patients 4–10 years F: 22 M: 32 -Analyzed: 54 patients	**Test group (TG**)**:** Professional removal and *S. salivarius M18* once a day for 3 months. **Control group** (**CG**)**:** Professional removal only.	T_0_: Baseline T_1_: 3 months T_2_ 6 months	*n* = 14 -Reason for attrition: * Patients with orthodontic treatment excluded (*n* = 3) * Patients with caries excluded (*n* = 7) * Excluded from analysis (antibiotic therapy during the study) (*n* = 4)	-Presence or absence of BSs -Oral hygiene index (OHI-s) by Greene and Vermillion	**Presence of BSs (number of patients (%)):** T_0_: TG: 0; CG: 0 T_1_: TG: 6 (21.2%); CG: 13 (50%) *p* = 0.02 * RR: 0.43 (IC 95%: 0.19 to 0.9); T_2_: TG: 9 (32.1%); CG: 14 (53.8%), *p* value = 0.1 RR: 0.60 (IC 95%: 0.31 to 1.16) **OHI-s values (mean + SD):** T_0_: TG: 1.95 ± 0.97; CG: 1.67 ± 1.15 *p* value = 0.3 T_1_: TG: 1.77 ± 1.23; CG: 1.81 ± 1.17 *p* value = 0.4 T_2_: TG: 1.06 ± 1.16; CG: 1.94 ± 1.27 *p* value = 0.02 *
**D’Errico G** **2021 (Italy)** [19]	Pilot randomized controlled trial	-Enrolled: 20 patients 8–24 years F: 12 M: 8 -Analyzed: 20 patients	**Test group:** Professional oral hygiene session and therapy with a probiotic food supply containing 2 types of *L. reuteri* for a duration of 2 months. **Control group:** Professional oral hygiene session.	T_0_: First visit T_1_: 30 days T_2_: 60 days	*n* = 0	-Modified Lobene index	**Modified Lobene index** (**mean value):** **Test group:** T_0_: 24.6%; T_1_: 4.7%; T_2_: 4.4% T_0_ = 24.6%; T_2_ = 4.4%; *p* < 0.05 **Control group:** T_0_: 39.0%; T_1_: 34.7%; T_2_: 49.3% **Statistical analysis of the mean Lobene index between the two groups:** T_1_: TG: 4.7%; CG: 34.7%, *p* < 0.05 MD −30.00% (95% CI: −36.19% to −23.81%) T_2_: TG: 4.4%; CG: 49.3%, *p* < 0.05 −44.90% (95% CI: −53.10% to −36.70%)
**Carelli M** **2023 (Italy)** [20]	Randomized controlled clinical trial	-26 patients F: 17 M: 9 -Analyzed	**Group without black stains** **Test group:** Toothpaste containing 1450 ppm sodium fluoride, enzymes, and salivary proteins twice a day for 14 weeks. **Control group:** Toothpaste with 1450 ppm amine fluoride twice a day for 14 weeks.	T_0_: Baseline T_1_: 14 weeks	*n* = 2 (from test group) Reason: poor compliance	-Prevalence of periodontal pathogens -Effects of enzymes and salivary proteins on periodontal pathogens -Shourie Index	***P. gingivalis* detection:** Subjects with BSs > subjects without BSs; *p* = 0.0129 * A.naeslundii detection: Subjects with BSs < subjects without BSs; *p* = 0.0064 * Effects of enzymes and salivary proteins on periodontal pathogens Test subjects showed a greater reduction in *P. gingivalis*, *A. naeslundii,* and *Veillonella* spp. in their saliva compared with control subjects. *p* > 0.05 **Shourie Index** T_1_: reduction in both test and control group, 8% and 12% average reduction, respectively, *p* > 0.05 20.45 (IC 95%: 4.68 to 36.22) 86% of BS patients presented a reduction in their Shourie Index Reduction in Shourie Index: patients using an electrical toothbrush > patients using a manual one, regardless of the toothpaste
**Wahba N** **2025 (Egypt)** [23]	In vitro and in vivo (non-randomized) study	-Enrolled: 9 patients 6–12 years -Analyzed: 9 patients	**One group:** Before and after probiotic treatment for 2 weeks Professional prophylaxis using ultrasonic scalers and polishing after sample collection and treatment with probiotics for 2 weeks	T_0_: Baseline T_1_: Post-probiotic treatment 2 weeks (In vivo study)	n = 0	-Bacterial load in saliva -Analysis of the presence of bacteria based on PCR amplification of the 16S rRNA with agarose gel electrophoresis (0.8%)	**In vivo study:** T_0_: Baseline presence of *A. actinomycetemcomitans* and *A.naeslundii* in all samples except 1 sample T_1_: A complete eradication of *A. actinomycetemcomitans* For *A. naeslundii*, mild significant reduction, except for a slight increase in 1 sample **Mean values of 16S rRNA of bacteria:** 16SrRNA of *A. actinomycetemcomitans*: T_0_: 0.48, T_1_: 0.00, *p* < 0.001 16SrRNA of *A. naeslundii*: T_0_: 0.67, T_1_: 0.45, *p* = 0.02; confirmed by PCR band intensity reduction

*T. forsythia: Tannerella forsythia*; *S. salivarius: Streptococcus salivarius*; *L. reuteri*: *Lactobacillus reuteri*; *P. gingivalis: Porphyromonas gingivalis*; *A. naeslundii*: *Actinomyces naeslundii*; *A. Actinomycetemcomitans*: *Aggregatibacter actinomycetemcomitans*; BS: black stain; CP: comeback period; RP: restoration period; aPDT: photodynamic therapy; OHI-s: oral hygiene index; * Statistically significant.

**Table 2:** Quality of the level of evidence by GRADE narrative assessment RCT: randomized controlled trials, NRCT: non-randomized controlled trials.

Interventions & Outcomes No. of Studies (Design)	Risk of Bias	Inconsistency	Indirectness	Imprecision	Publication Bias	Overall Certainty (GRADE)	Comments
**Probiotics ** Black stain index 3 RCTs	Very Serious	Not Serious	Not serious	Serious	Undetected	VERY LOW ⨁◯◯◯	All three studies reported a reduction in recurrence in the test groups. However, the high risk of bias and small sample sizes reduced the certainty of the evidence for this outcome.
Bacterial load 1 NRCT	Serious	Not serious	Serious	Serious	Undetected	VERY LOW ⨁◯◯◯	One study reported a statistically significant reduction in bacterial load. However, the certainty of the evidence was downgraded due to serious risk of bias, serious indirectness because bacterial load was assessed as a surrogate outcome, and imprecision resulting from the small sample size.
**Mouthwash** Black stain index 1 NRCT	Serious	Not serious	Not serious	Serious	Undetected	VERY LOW ⨁◯◯◯	The study suggested a reduction in the Pigmented Plaque Index; however, no statistical significance was reported. The certainty of evidence was downgraded due to serious risk of bias and serious imprecision.
Bacterial load 1 RCT 1 NRCT	Serious	Not Serious	Serious	Serious	Undetected	VERY LOW ⨁◯◯◯	Both studies reported a reduction in bacterial load, although only one study reported statistical significance. Certainty of evidence was downgraded due to risk of bias and serious imprecision related to the small sample sizes.
**Enzyme-based toothpaste** Black stain index 1 RCT	Serious	Not serious	Not serious	Serious	Undetected	LOW ⨁⨁◯◯	The certainty of evidence was downgraded due to serious risk of bias and serious imprecision related to the small sample sizes.
Bacterial load 1 RCT	Serious	Not serious	Serious	Serious	Undetected	VERY LOW ⨁◯◯◯	The author reported a reduction in bacterial load; however, the reduction was not statistically significant in the study.
**Photodynamic therapy** Black stain index 1 NRCT	Serious	Not serious	Not serious	Serious	Undetected	VERY LOW ⨁◯◯◯	No significant effect on the time to reappearance of bacterial black stains was observed.
Bacterial load 1 NRCT	Serious	Not serious	Serious	Serious	Undetected	VERY LOW ⨁◯◯◯	No statistically significant reduction in bacterial load was observed. The certainty of evidence was downgraded due to serious risk of bias and serious imprecision.

## Data Availability

No new data were created or analyzed in this study.

## Supplementary Materials

The following supporting information can be downloaded at https://www.mdpi.com/article/10.3390/dj14100638/s1. Table S1: List of excluded studies with reasons; Table S2: PRISMA 2020 checklist

## Appendix A

**Table A1:** Electronic databases and search strategy (30 April 2026).

Database (n) or Registry		Search Strategy #1 AND #2
WoS (*n* = 184) Web of Science Core Collection [11]	#1	TS = “black stain*” OR “dental black stain*” OR “extrinsic black stain*” OR “chromogenic stain” OR “chromogenic bacteria”
	#2	TS = treatment OR therapy OR removal OR management OR scaling OR polishing OR prophylaxis OR probiotic* OR toothpaste* OR mouthwash* OR “chemical agent*” OR photodynamic OR “photodynamic therapy” OR “PDT” OR “photoactivated disinfection” OR “photochemotherapy”
	#1 AND #2	(TS = (“black stain*” OR “dental black stain*” OR “extrinsic black stain*” OR “chromogenic stain” OR “chromogenic bacteria”)) AND TS = (treatment OR therapy OR removal OR management OR scaling OR polishing OR prophylaxis OR probiotic* OR toothpaste* OR mouthwash* OR “chemical agent*” OR photodynamic OR “photodynamic therapy” OR “PDT” OR “photoactivated disinfection” OR “photochemotherapy”)
Scopus (*n* = 225) https://www.scopus.com	#1	TITLE-ABS-KEY “black stain*” OR “dental black stain*” OR “extrinsic black stain*” OR “chromogenic stain” OR “chromogenic bacteria”
	#2	TITLE-ABS-KEY treatment OR therapy OR removal OR management OR scaling OR polishing OR prophylaxis OR probiotic* OR toothpaste* OR mouthwash* OR “chemical agent*” OR photodynamic OR “photodynamic therapy” OR “PDT” OR “photoactivated disinfection” OR “photochemotherapy”
	#1 AND #2	(TITLE-ABS-KEY (“black stain*” OR “dental black stain*” OR “extrinsic black stain*” OR “chromogenic stain” OR “chromogenic bacteria”) AND TITLE-ABS-KEY (treatment OR therapy OR removal OR management OR scaling OR polishing OR prophylaxis OR probiotic* OR toothpaste* OR mouthwash* OR “chemical agent*” OR photodynamic OR “photodynamic therapy” OR “PDT” OR “photoactivated disinfection” OR “photochemotherapy”))
PubMed (*n* = 216) https://pubmed.ncbi.nlm.nih.gov/	#1	“black stain*” OR “dental black stain*” OR “extrinsic black stain*” OR “chromogenic stain” OR “chromogenic bacteria”
	#2	treatment OR therapy OR removal OR management OR scaling OR polishing OR prophylaxis OR probiotic* OR toothpaste* OR mouthwash* OR “chemical agent*” OR photodynamic OR “photodynamic therapy” OR “PDT” OR “photoactivated disinfection” OR “photochemotherapy”
	#1 AND #2	(“black stain*” OR “dental black stain*” OR “extrinsic black stain*” OR “chromogenic stain” OR “chromogenic bacteria”) AND (treatment OR therapy OR removal OR management OR scaling OR polishing OR prophylaxis OR probiotic* OR toothpaste* OR mouthwash* OR “chemical agent*” OR photodynamic OR “photodynamic therapy” OR “PDT” OR “photoactivated disinfection” OR “photochemotherapy”) Sort by: Publication Date (“black stain*”[All Fields] OR “dental black stain*”[All Fields] OR “extrinsic black stain*”[All Fields] OR “chromogenic stain”[All Fields] OR “chromogenic bacteria”[All Fields]) AND (“therapeutics”[MeSH Terms] OR “therapeutics”[All Fields] OR “treatments”[All Fields] OR “therapy”[MeSH Subheading] OR “therapy”[All Fields] OR “treatment”[All Fields] OR “treatment s”[All Fields] OR (“therapeutics”[MeSH Terms] OR “therapeutics”[All Fields] OR “therapies”[All Fields] OR “therapy”[MeSH Subheading] OR “therapy”[All Fields] OR “therapy s”[All Fields] OR “therapies”[All Fields]) OR (“removability”[All Fields] OR “removal”[All Fields] OR “removals”[All Fields] OR “remove”[All Fields] OR “removed”[All Fields] OR “removement”[All Fields] OR “remover”[All Fields] OR “removers”[All Fields] OR “removes”[All Fields] OR “removing”[All Fields]) OR (“manage”[All Fields] OR “managed”[All Fields] OR “management s”[All Fields] OR “managements”[All Fields] OR “manager”[All Fields] OR “manager s”[All Fields] OR “managers”[All Fields] OR “manages”[All Fields] OR “managing”[All Fields] OR “management”[All Fields] OR “organization and administration”[MeSH Terms] OR (“organization”[All Fields] AND “administration”[All Fields]) OR “organization and administration”[All Fields] OR “management”[All Fields] OR “disease management”[MeSH Terms] OR (“disease”[All Fields] AND “management”[All Fields]) OR “disease management”[All Fields]) OR (“scale”[All Fields] OR “scale s”[All Fields] OR “scaled”[All Fields] OR “scales”[All Fields] OR “scaling”[All Fields] OR “scalings”[All Fields]) OR (“polished”[All Fields] OR “polisher”[All Fields] OR “polishers”[All Fields] OR “polishing”[All Fields] OR “polishings”[All Fields]) OR (“prevention and control”[MeSH Subheading] OR (“prevention”[All Fields] AND “control”[All Fields]) OR “prevention and control”[All Fields] OR “prophylaxis”[All Fields] OR “prophylaxes”[All Fields] OR “prophylaxy”[All Fields]) OR “probiotic*”[All Fields] OR “toothpaste*”[All Fields] OR “mouthwash*”[All Fields] OR “chemical agent*”[All Fields] OR (“photodynamic”[All Fields] OR “photodynamical”[All Fields] OR “photodynamically”[All Fields] OR “photodynamics”[All Fields]) OR “photodynamic therapy”[All Fields] OR “PDT”[All Fields] OR “photoactivated disinfection”[All Fields] OR “photochemotherapy”[All Fields])
Cochrane (*n* = 39) https://www.cochranelibrary.com/advanced-search accessed on 28 August 2026	#1	TITLE ABSTRACT KEYWORD “black stain*” OR “dental black stain*” OR “extrinsic black stain*” OR “chromogenic stain” OR “chromogenic bacteria”
	#2	TITLE-ABSTRACT-KEYWORD treatment OR therapy OR removal OR management OR scaling OR polishing OR prophylaxis OR probiotic* OR toothpaste* OR mouthwash* OR “chemical agent*” OR photodynamic OR “photodynamic therapy” OR “PDT” OR “photoactivated disinfection” OR “photochemotherapy”
	#1 AND #2	(“Black stain*” OR “dental black stain*” OR “extrinsic black stain*” OR “chromogenic stain” OR “chromogenic bacteria”) AND (treatment OR therapy OR removal OR management OR scaling OR polishing OR prophylaxis OR probiotic* OR toothpaste* OR mouthwash* OR “chemical agent*” OR photodynamic OR “photodynamic therapy” OR “PDT” OR “photoactivated disinfection” OR “photochemotherapy”)
Dentistry & Oral Sciences Source (*n* = 45) https://research.ebsco.com/c/7oqckh/search accessed on 28 August 2026	#1	“black stain*” OR “dental black stain*” OR “extrinsic black stain*” OR “chromogenic stain” OR “chromogenic bacteria”
	#2	treatment OR therapy OR removal OR management OR scaling OR polishing OR prophylaxis OR probiotic* OR toothpaste* OR mouthwash* OR “chemical agent*” OR photodynamic OR “photodynamic therapy” OR “PDT” OR “photoactivated disinfection” OR “photochemotherapy”
Google Scholar (n = first 100 results) https://scholar.google.com/	#1	“black stain*” OR “dental black stain*” OR “extrinsic black stain*” OR “chromogenic stain” OR “chromogenic bacteria”
	#2	treatment OR therapy OR removal OR management OR scaling OR polishing OR prophylaxis OR probiotic* OR toothpaste* OR mouthwash* OR “chemical agent*” OR photodynamic OR “photodynamic therapy” OR “PDT” OR “photoactivated disinfection” OR “photochemotherapy”
Opengrey (*n* = 0) https://opengrey.eu/	#1	“black stain*” OR “dental black stain*” OR “extrinsic black stain*” OR “chromogenic stain” OR “chromogenic bacteria”
	#2	treatment OR therapy OR removal OR management OR scaling OR polishing OR prophylaxis OR probiotic* OR toothpaste* OR mouthwash* OR “chemical agent*” OR photodynamic OR “photodynamic therapy” OR “PDT” OR “photoactivated disinfection” OR “photochemotherapy”
ISRCTN registry (*n* = 0) https://www.isrctn.com/	#1	“black stain*” OR “dental black stain*” OR “extrinsic black stain*” OR “chromogenic stain” OR “chromogenic bacteria”
	#2	treatment OR therapy OR removal OR management OR scaling OR polishing OR prophylaxis OR probiotic* OR toothpaste* OR mouthwash* OR “chemical agent*” OR photodynamic OR “photodynamic therapy” OR “PDT” OR “photoactivated disinfection” OR “photochemotherapy”
Clinicaltrial.gov (*n* = 6) https://clinicaltrials.gov/	#1	“black stain*” OR “dental black stain*” OR “extrinsic black stain*” OR “chromogenic stain” OR “chromogenic bacteria”
	#2	treatment OR therapy OR removal OR management OR scaling OR polishing OR prophylaxis OR probiotic* OR toothpaste* OR mouthwash* OR “chemical agent*” OR photodynamic OR “photodynamic therapy” OR “PDT” OR “photoactivated disinfection” OR “photochemotherapy”

*n*—number of hits, WoS—Web of Science Core Collection, CENTRAL—Cochrane Central Register of Controlled Trials. TS—Topic (article title, abstract, and keywords), # query line; * Truncation/wildcard operator to include word variations (singular/plural forms).
