# Supplementary material for: Alternative Strategies for Managing Dental Black Stains: A Systematic Review
Source: Dent J (Basel). 2026 Oct 4;14(10):638. doi: 10.3390/dj14100638 (PMC13703232; doi:10.3390/dj14100638)
Supplement: Supplementary file 1 [file dentistry-14-00638-s001.zip › dentistry-4373019-Table S1.pdf]

List of excluded studies with reasons.

Author	Article title	Reason for exclusion	Study status
Mohamed Farouk Rashed	Effect of Herbal Toothpaste Versus Conventional One on Black Stains	Reports not retrieved. We attempted to contact the author by email, but received no response. 04/04/2026	**Study Completion (Actual)**<br>2025-03-01
Ramesh Kumar	Effect of oil pulling in preventing black stains on teeth among adults	Reports not retrieved. We attempted to contact the author by email, but received no response.04/04/2026	Not yet recruiting
Sanjana V	Can laser therapy help reduce and prevent the black stains on patients complaining of frequent recurrence when used along with cleaning	Reports not retrieved. We attempted to contact the author by email, but received no response.<br>04/04/2026	Not Yet Recruiting
Andrea Scribante	Black Stains' Epidemiology, Clinical Evaluation and Dietary Habits: a Clinical Trial	Reports not retrieved. We attempted to contact the author by email, but received no response.<br>04/04/2026	**Study Completion (Actual)**<br>2022-01-16
María del Mar Jovani-Sancho	Air Polishers With Erythritol in the Treatment of Dental Black Stain of Bacterial Origin	Reports not retrieved. We attempted to contact the author by email, but received no response<br>.04/04/2026	Enrolling
Veronica Veses	Effectiveness of Antimicrobial Photodynamic Therapy for the Elimination of Dental Black Stain: A Randomized Clinical Trial	Reports not retrieved. We attempted to contact the author by email; she answered, “We concluded the clinical trial and found no relevant differences between the control and the intervention time.”	**Study Completion (Actual)**<br>2020-01-30
Sruthy Prathap* and Prathap MS	Occurrence of Black Chromogenic Stains and its Association with Oral Hygiene of Patients	No adjunctive treatment was evaluated; only recurrence following scaling and polishing was assessed.	
